# Biological Control Activities of Rice-Associated *Bacillus* sp. Strains against Sheath Blight and Bacterial Panicle Blight of Rice

**DOI:** 10.1371/journal.pone.0146764

**Published:** 2016-01-14

**Authors:** Bishnu K. Shrestha, Hari Sharan Karki, Donald E. Groth, Nootjarin Jungkhun, Jong Hyun Ham

**Affiliations:** 1 Department of Plant Pathology and Crop Physiology, Louisiana State University Agricultural Center, Baton Rouge, Louisiana, 70803, United States of America; 2 Rice Research Station, Louisiana State University Agricultural Center, Rayne, Louisiana, 70578, United States of America; 3 Chiang Rai Rice Research Center, Bureau of Rice Research and Development, Rice Department, 474 Moo 9, Phaholyothin Rd., Muang Phan, Phan, Chiang Rai, 57120, Thailand; Loyola University Chicago, UNITED STATES

## Abstract

Potential biological control agents for two major rice diseases, sheath blight and bacterial panicle blight, were isolated from rice plants in this study. Rice-associated bacteria (RABs) isolated from rice plants grown in the field were tested for their antagonistic activities against the rice pathogens, *Rhizoctonia solani* and *Burkholderia glumae*, which cause sheath blight and bacterial panicle blight, respectively. Twenty-nine RABs were initially screened based on their antagonistic activities against both *R*. *solani* and *B*. *glumae*. In follow-up retests, 26 RABs of the 29 RABs were confirmed to have antimicrobial activities, but the rest three RABs did not reproduce any observable antagonistic activity against *R*. *solani* or *B*. *glumae*. According to16S rDNA sequence identity, 12 of the 26 antagonistic RABs were closest to *Bacillus amyloliquefaciens*, while seven RABs were to *B*. *methylotrophicus* and *B*, *subtilis*, respectively. The 16S rDNA sequences of the three non-antagonistic RABs were closest to *Lysinibacillus sphaericus* (RAB1 and RAB12) and *Lysinibacillus macroides* (RAB5). The five selected RABs showing highest antimicrobial activities (RAB6, RAB9, RAB16, RAB17S, and RAB18) were closest to *B*. *amyloliquefaciens* in DNA sequence of 16S rDNA and *gyrB*, but to *B*. *subtilis* in that of *recA*. These RABs were observed to inhibit the sclerotial germination of *R*. *solani* on potato dextrose agar and the lesion development on detached rice leaves by artificial inoculation of *R*. *solani*. These antagonistic RABs also significantly suppressed the disease development of sheath blight and bacterial panicle blight in a field condition, suggesting that they can be potential biological control agents for these rice diseases. However, these antagonistic RABs showed diminished disease suppression activities in the repeated field trial conducted in the following year probably due to their reduced antagonistic activities to the pathogens during the long-term storage in -70C, suggesting that development of proper storage methods to maintain antagonistic activity is as crucial as identification of new biological control agents.

## Introduction

A diverse range of microorganisms dwell in various parts of a plant, causing detrimental, neutral or beneficial effects on plant health [1–3]. Some of plant-inhabiting microorganisms can suppress plant diseases through competition, predation or antagonism against plant pathogens, or through induction of plant defense systems [4, 5]. Antagonistic bacteria isolated from plant surface, soil and rhizosphere have been extensively used to control major crop diseases caused by various fungal and bacterial diseases [6, 7]. Those microorganisms can be used alone or in combination with other chemical or biological control agents for various crop diseases [1–3, 6, 8, 9] [10, 11].

Sheath blight is one of the most economically important rice diseases worldwide, which is caused by the fungal pathogen *Rhizoctonia solani*. Typical symptoms of this disease are oval to irregular lesions with grayish inner and dark brown margin colors on rice sheath and leaf blades. *R*. *solani* is a soilborne pathogen having a broad host range including rice and soybean. Epidemics of sheath blight occur throughout the temperate and tropical rice-growing regions. High nitrogen rates and plant density provide favorable microclimate conditions for the development of sheath blight during early heading and grain-filling stages [12]. Common practices for the management of sheath blight include crop rotation, fertilizer management, planting disease-tolerant varieties, and fungicide application. However, rice cultivars having vertical (or complete) resistance to the disease are not available to cultivate, and crop rotation will not assure effective management of the disease because the fungus can survive for a long period of time in the form of sclerotia, a primary source of inoculum that overwinters in soil and plant debris. Various fungicides are being used to control the disease [13–15], however, fungicide application increases the cost of cultivation and the risk of the emergence of fungicide-resistant pathogens [16].

Bacterial panicle blight, which is caused by the Gram-negative bacterial pathogens *Burkholderia glumae* and *B*. *gladioli*, is another important rice disease in many rice-growing regions around the world [17–20]. The major symptoms of this disease include panicle discoloration, grain rot, and sterile florets. Prolonged high night-temperatures during the heading and flowering stages favor the outbreaks of bacterial panicle blight [19, 21, 22]. *B*. *glumae*, the chief causal organism of bacterial panicle blight [23], is a seed-borne bacterium and produces the yellow-colored phytotoxin, toxoflavin, as a major virulence factor [24]. Despite the economic importance of bacterial panicle blight, there is few control measures for this disease. There is no known complete resistance for this disease and only a few partially resistant varieties are commercially available [25, 26]. Oxolinic acid is the only known commercial chemical agent for controlling this disease [27]. However, this chemical is not registered for agricultural purposes in some countries including the United States [19], and natural occurrence of oxolinic acid-resistant strains limits the usage of this chemical [28].

In this study, rice-associated bacteria (RABs) showing various levels of antagonistic activities against *R*. *solani* and *B*. *glumae* were isolated and identified based on their DNA sequences of 16S rDNA, *gyrA*, and *recA*. Furthermore, *in vivo* antagonistic activities and disease control efficacies in the rice field for sheath blight and bacterial panicle blight, as well as rice growth promoting activities, were tested with five selected RABs showing highest levels of antimicrobial activities.

## Results

### Isolation of RAB Strains Showing Antimicrobial Activities against *R*. *solani* and *B*. *glumae*

**Fig 1:**
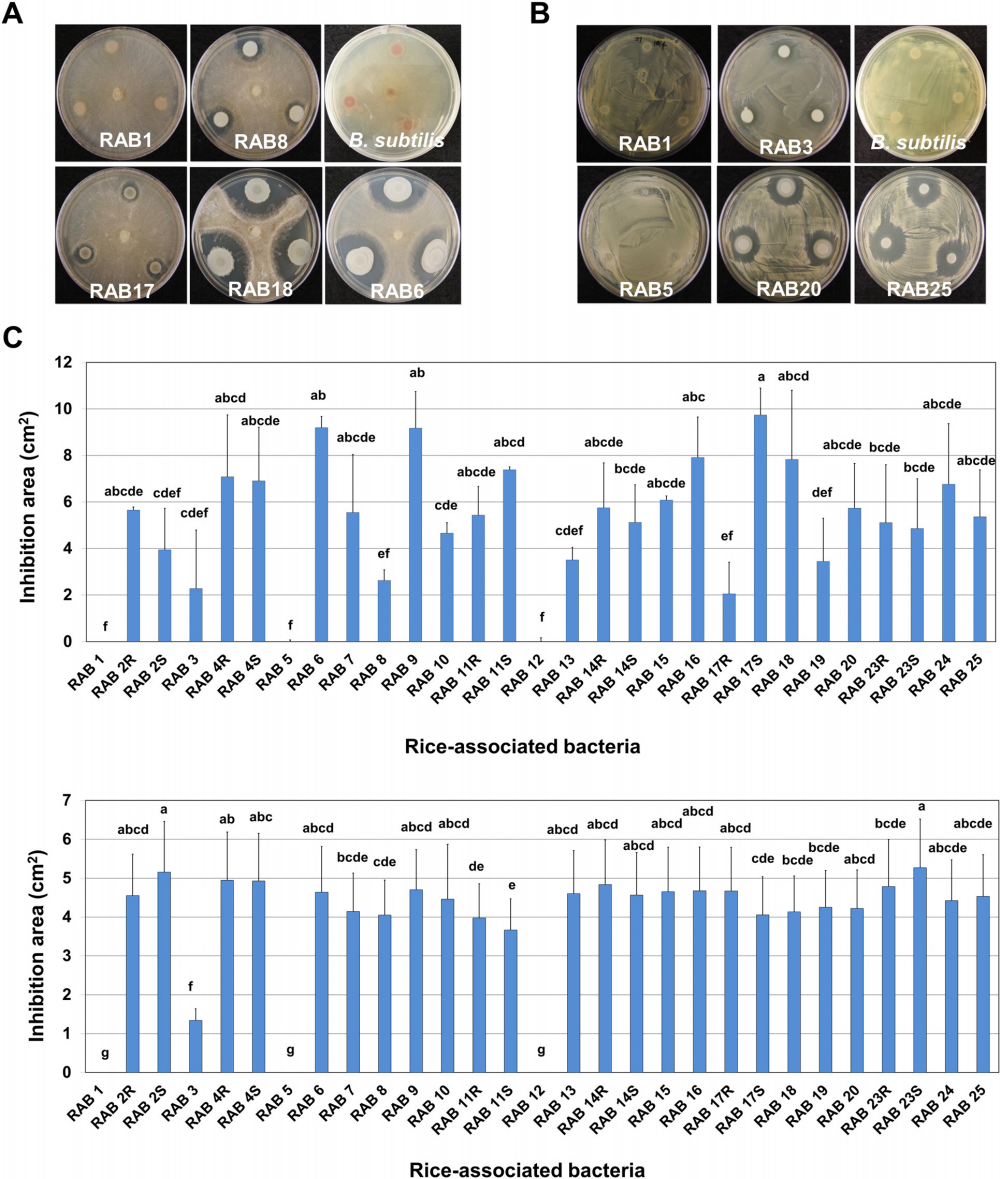
Antibacterial and antifungal activities of RABs revealed by dual culture experiments. The bacterial suspension of each RAB was spotted after *Burkholderia glumae* cells were spread-plated, or a mycelial plug of *Rhizoctonia solani* was placed on the center of a PDA plate. The plates of dual culture were incubated at 25C for 72 h in the dark. (A) Selected plates of dual culture showing antifungal activities of RABs against *R*. *solani*. (B) Selected plates of dual culture showing antibacterial activities of RABs against *B*. *glumae*. (C) Antifungal (upper graph) and antibacterial (lower graph) activities of RABs against *R*. *solani* and *B*. *glumae*, respectively. Each error bar indicates standard deviation from three replications. The letter above each column indicates significant difference from other data with *P <* 0.0001 based on Tukey’s test and each error bar indicates standard deviation from three replications.

Twenty-nine RAB strains (RABs) out of the total 127 RABs tested were initially screened based on their antimicrobial activities against both *R*. *solani* and *B*. *glumae*. Through repeated follow-up experiments with the 29 RABs, 26 RABs were confirmed to have antagonistic activities, however, the remaining three RABs (RAB1, RAB5 and RAB12) did not show any antifungal or antibacterial activities again (Fig 1). According to the sizes of the inhibition zones, the antibacterial activities of the 26 active RABs were overall less variable than their antifungal activities, except that RAB3 showed a much lower antibacterial activity compared to other antagonistic RABs (Fig 1C and 1D). Across the 26 antagonistic RABs, RAB2S, RAB3, RAB8, RAB13, RAB17R and RAB19 showed relatively lower antifungal activities, while RAB6, RAB9 and RAB17S showed relatively higher antifungal activities against *R*. *solani* (Fig 1C).

### Identification of RABs

**Fig 2:**
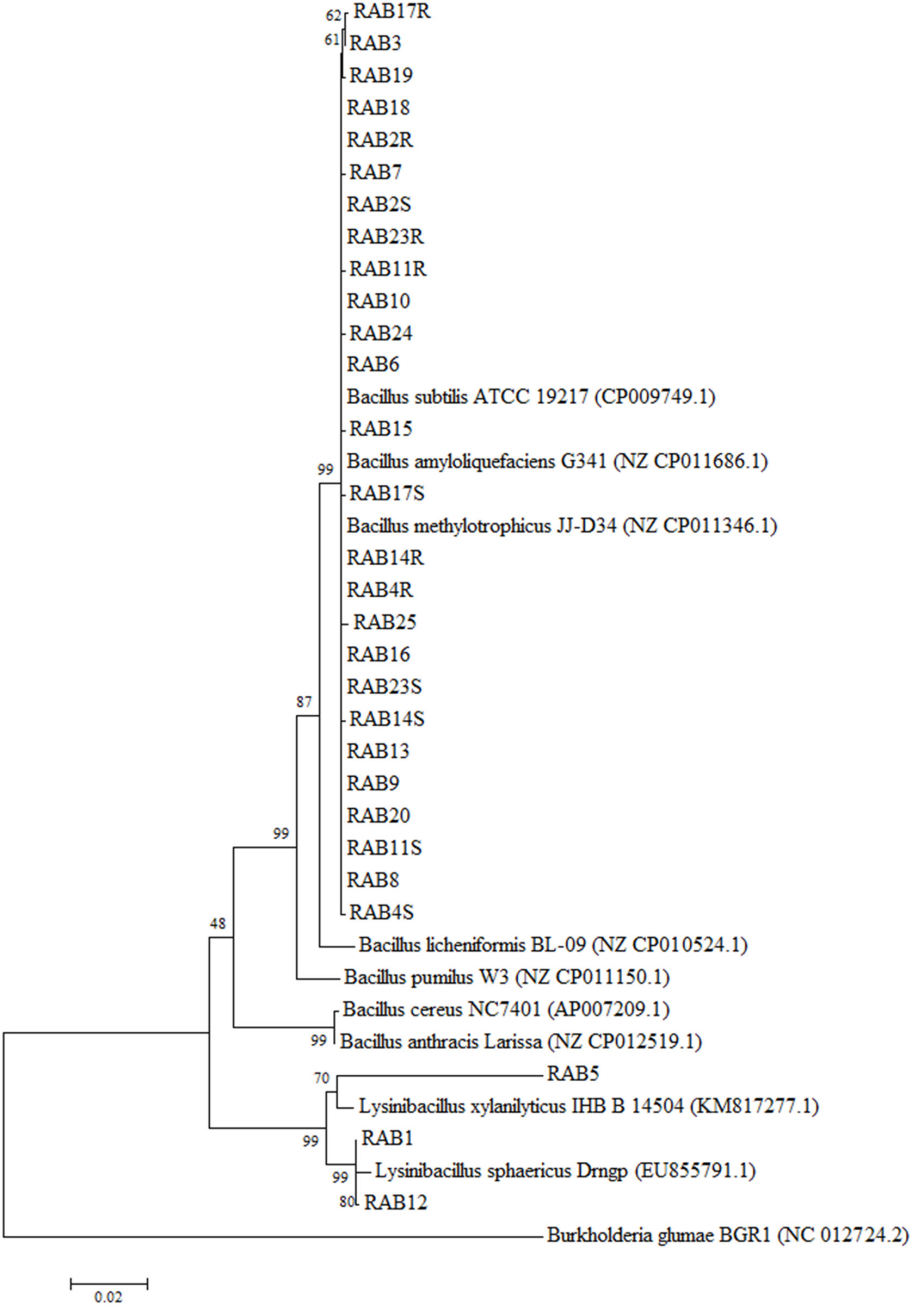
Phylogenetic tree based on 16S rDNA sequences of the 29 rice-associated bacteria (RABs) and other *Bacillus* species from GenBank nucleotide database. GenBank accession numbers for the sequences obtained from the database are given in parentheses. The tree was generated using neighbor-joining method, and genetic distances were calculated using the Kimura 2-parameter method using MEGA5 [76]. Numbers at nodes indicate percentage of occurrence in 1000 bootstrapping. The bar at the bottom of the tree indicates the scale for genetic distances. *Burkholderia glumae* 336gr-1 was used as an out-group control.

In the Ryu’s 3% KOH tests, all the 29 RABs tested were determined to be Gram-positive along with the control bacterium *B*. *subtilis*, while *B*. *glumae* cells used as the Gram-negative control bacterium exhibited typical mucous thread of genomic DNA (Table 1). BLAST searches of the 16S rDNA sequences against the NCBI database revealed the bacterial species showing the highest sequence identity to each RAB (Table 1). In this approach, 12 of the 26 antagonistic RABs were closest to *B*. *amyloliquefaciens*, and seven of the remaining 14 RABs were to *B*. *methyllotrophicus* and *B*. *subtilis*, respectively (Table 1). The three non-antagonistic RABs were closest to *Lysinibacillus sphaericus* (RAB1, RAB12) and *Lysinibacillus macroides* (RAB5) (Table 1). Consistent with the BLAST search results shown in Table 1, all the 26 RABs showing antimicrobial activities were clustered in a same major clade along with *B*. *amyloliquefaciens*, *B*. *methylotrophicus* and *B*. *subtilis*, while the three non-antagonistic RABs (RAB1, RAB5, and RAB12) were clustered as a separate clade along with *Lysinibacillus sphaericus* (RAB1 and RAB12) or *L*. *xylanilyticus* (RAB5) (Fig 2). Among the species of *Bacillus* included in this cluster analysis, *B*. *anthracis*, *B*. *cereus*, *B*. *pumilus*, and *B*. *licheniformis* were grouped as separate clades from the antagonistic RABs, indicating that the RABs in this study are distantly related to these species (Fig 2). However, these species were exhibited to be more closely related to the antagonistic RABs than to the non-antagonistic RABs (Fig 2).

**Table 1:** Gram reactions and predicted bacterial species of rice associated bacteria (RABs).

RAB strain	Closest species in GenBank based on 16S rDNA sequence	Strain name	Accession number	Query coverage (%)	Sequence Identity (%)	E-value	Gram reaction^a^
**RAB1**	*Lysinibacillus sphaericus*	C5	KF523303.1	100	100	0.0	Gram +
**RAB2R**	*Bacillus methylotropicus*	BHR3P2B1S	KJ567098.2	100	100	0.0	Gram +
**RAB2S**	*Bacillus subtilis*	J-18	KJ934378.1	100	100	0.0	Gram +
**RAB3**	*Bacillus amyloliquefaciens*	Y-32-1	KT833128.1	100	99	0.0	Gram +
**RAB4R**	*Bacillus methylotrophicus*	KD3	KR855693.1	100	100	0.0	Gram +
**RAB4S**	*Bacillus subtillis*	RSS-1	KR086418.1	100	99	0.0	Gram +
**RAB5**	*Lysinibacillus macroides*	Y-4B	JX502177.1	100	100	0.0	Gram +
**RAB6**	*Bacillus amyloliquefaciens*	G341	CP011686.1	100	100	0.0	Gram +
**RAB7**	*Bacillus methylotrophicus*	LD34	KR855694.1	100	99	0.0	Gram +
**RAB8**	*Bacillus methylotrophicus*	LD34	KR855694.1	100	100	0.0	Gram +
**RAB9**	*Bacillus amyloliquefaciens*	G341	CP011686.1	100	100	0.0	Gram +
**RAB10**	*Bacillus amyloliquefaciens*	G341	CP011686.1	100	100	0.0	Gram +
**RAB11R**	*Bacillus methylotrophicus*	LD34	KR855694.1	100	99	0.0	Gram +
**RAB11S**	*Bacillus amyloliquefaciens*	RJ2	KR090589.1	100	100	0.0	Gram +
**RAB12**	*Lysinibacillus sphaericus*	C5	KF523303.1	99	99	0.0	Gram +
**RAB13**	*Bacillus subtilis*	YB-6	KT377368.1	100	100	0.0	Gram +
**RAB14R**	*Bacillus subtilis*	J-18	KJ934378.1	100	100	0.0	Gram +
**RAB14S**	*Bacillus subtilis*	J-18	KJ934378.1	100	99	0.0	Gram +
**RAB15**	*Bacillus amyloliquefaciens*	RJ2	KR090589.1	100	99	0.0	Gram +
**RAB16**	*Bacillus amyloliquefaciens*	MB2-A2	KT935664.1	100	100	0.0	Gram +
**RAB17R**	*Bacillus methylotrophicus*	RJ2	KR090589.1	100	99	0.0	Gram +
**RAB17S**	*Bacillus amyloliquefaciens*	RJ2	KR090589.1	100	99	0.0	Gram +
**RAB18**	*Bacillus amyloliquefaciens*	G341	CP011686.1	100	100	0.0	Gram +
**RAB19**	*Bacillus amyloliquefaciens*	MB2-A41	KT935670.1	100	99	0.0	Gram +
**RAB20**	*Bacillus amyloliquefaciens*	RJ2	KR090589.1	100	100	0.0	Gram +
**RAB23R**	*Bacillus subtilis*	RSS-1	KR086418.1	100	100	0.0	Gram +
**RAB23S**	*Bacillus subtilis*	BS-S3	AY583216.1	99	100	0.0	Gram +
**RAB24**	*Bacillus methylotrophicus*	LD34	KR855694.1	100	99	0.0	Gram +
**RAB25**	*Bacillus amyloliquefaciens*	Zy25	JX491658.1	97	99	0.0	Gram +

^a^: Gram reaction was determined by the Ryu’s KOH test [66, 67].

### Biological Activities of Five Selected Antagonistic RABs on *R*. *solani*

**Fig 3:**
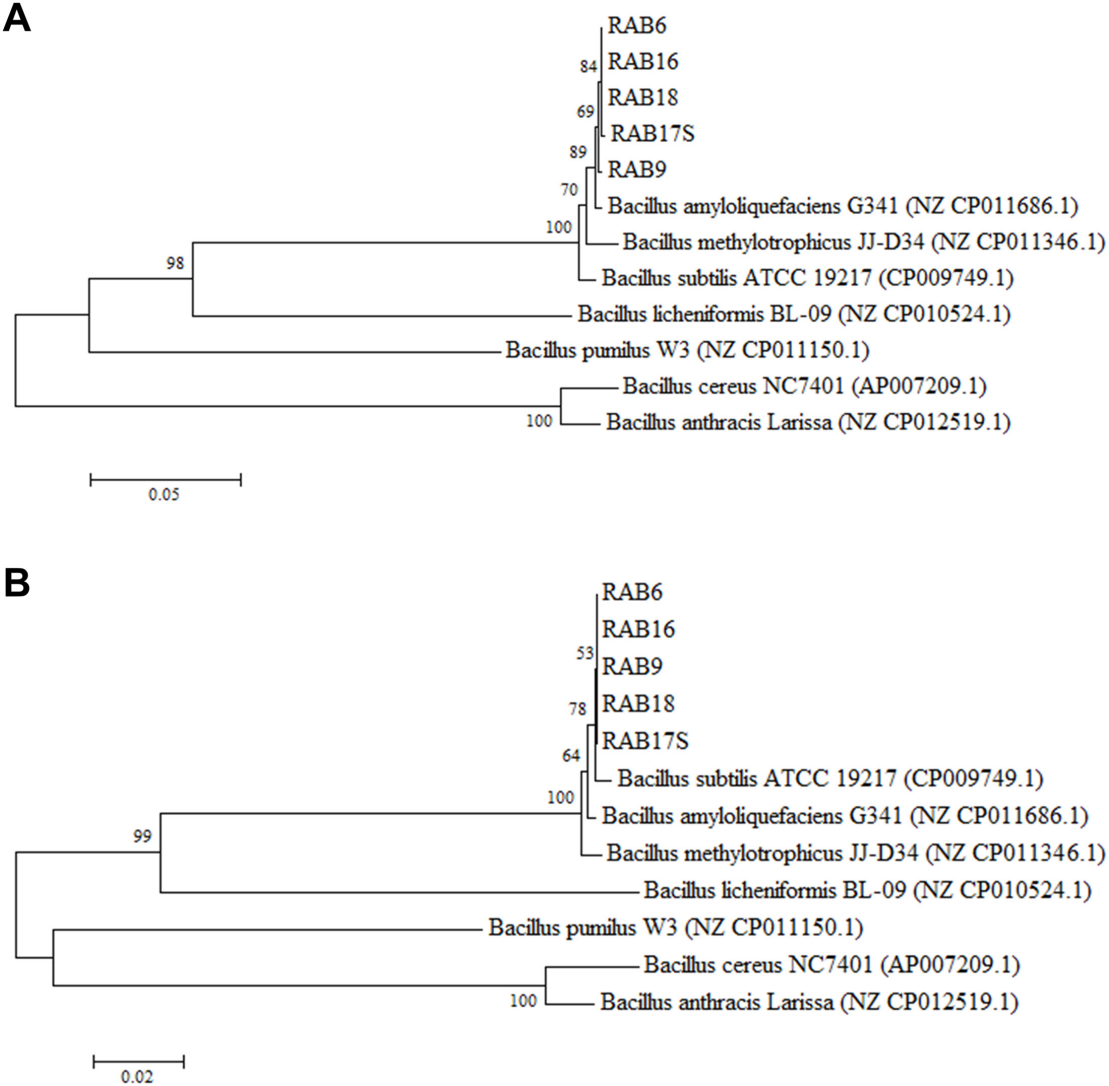
Phylogenetic tree based on *gyrB* (A) and *recA* (B) sequences of the five rice-associated bacteria (RABs) and other *Bacillus* species from GenBank nucleotide database. GenBank accession numbers for the sequences obtained from the database are given in parentheses. The tree was generated using neighbor-joining method, and genetic distances were calculated using the Kimura 2-parameter method using MEGA5 [76]. Numbers at nodes indicate percentage of occurrence in 1000 bootstrapping. The bar at the bottom of the tree indicates the scale for genetic distances. *Burkholderia glumae* 336gr-1 was used as an out-group control.

**Fig 4:**
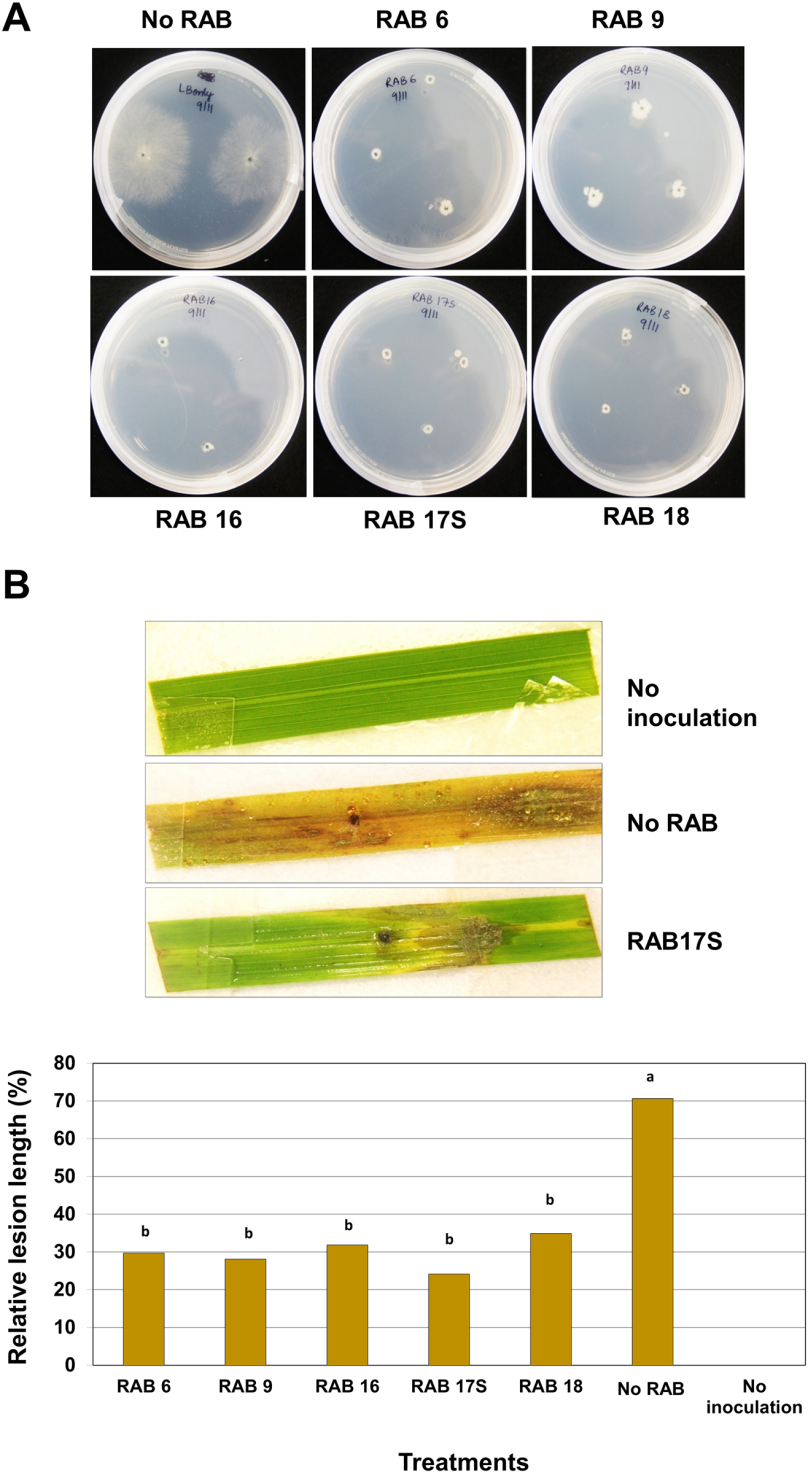
Inhibitory activities of antagonistic RABs on *Rhizoctonia solani*. (A) Inhibition of sclerotial germination by RABs. Overnight cultured sclerotia with RAB inoculum in test tube were grown on PDA media and incubated at 25°C for 72 h. Sclerotia cultured only in LB broth were used as control. Sclerotial germination was inhibited by all RABs inoculum except in the control where the mycelial growth can be observed. (B) Inhibition of sheath blight lesion development by pretreatment of RABs: Examples of detached leaf assay revealing inhibitory activities of RABs against lesion development by *R*. *solani*. A detached rice leaf pretreated with a RAB was placed on paper towel moisturized with sterilized ddH_2_O and a sclerotium of *R*. *solani* was inoculated on the center of the detached leaf. Petri-dishes containing the inoculated rice leaves were incubated at 25°C for 7 d with 12 h of light period per day (upper); and Inhibition of sheath blight lesion development by the five selected RABs that showed highest antifungal activities in the dual-culture assay (lower). Each column of the graph represents the average of three replications and the letter above each column indicates significant difference with *P* < 0.05, which was determined by Tukey’s test.

Five antagonistic RABs (RAB6, RAB9, RAB16, RAB17S, and RAB18) were selected for further characterization in other biological activities on *R*. *solani*. All of the selected RABs appeared to be *B*. *amyloliquefaciens* according to the phylogenetic analysis of their 16S rDNA (Fig 2) and *gyrB* (Fig 3A) sequences. However, these five RABs were more closely related to *B*. *subtilis* ATCC19217 than to *B*. *amyloliquefaciens* G341 in terms of the DNA sequences for *recA* (Fig 3B), in which only two bases were different between the two bacterial species. Germination of *R*. *solani* sclerotia was completely inhibited by the five selected RABs (Fig 4A). In detached leaf assays, all the five selected RABs significantly restricted the development of sheath blight lesions (Fig 4B).

### Biological Control Activities of the Selected Antagonistic RABs

**Fig 5:**
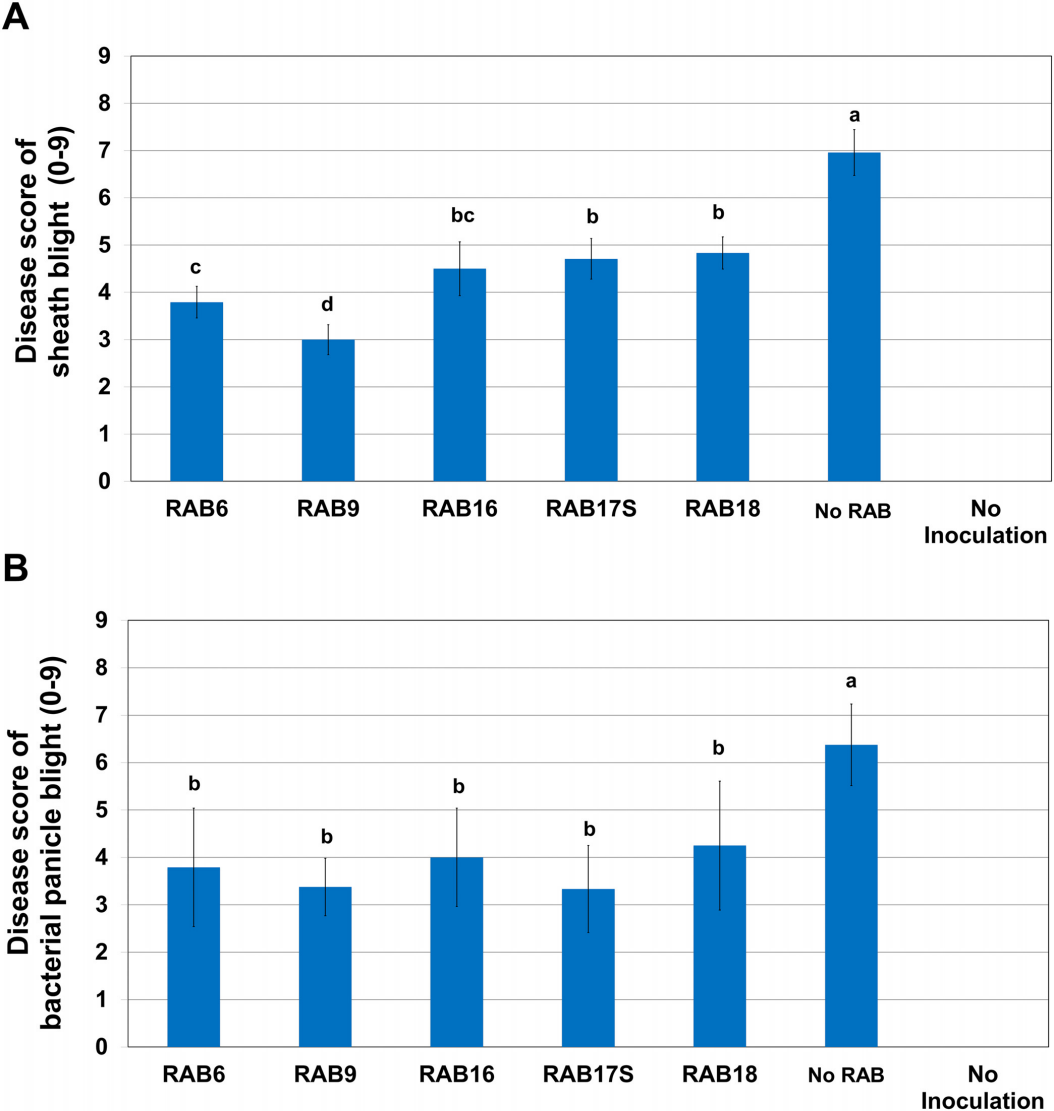
Biological control activities of selected RABs in the field obtained from the field trial in 2012. (A) Suppression of sheath blight by RABs. *R*. *solani* was inoculated 24 h after the treatment of RABs at the tillering stage of rice plants (cv. Bengal). Rice plants treated with water instead of a RAB and those without inoculation of *R*. *solani* were included as controls. Disease severity was rated 27 days after inoculation based on a 0 to 9 scale scheme [79]. Each error bar indicates standard error from three replications and the letters above individual columns indicate significant differences with *P* < 0.05 according to Dunn’s test. (B) Suppression of bacterial panicle blight by RABs. *B*. *glumae* was inoculated 24 h after the treatment of RABs at the 30% heading stage of rice plants (cv. Bengal). Rice plants treated with water instead of a RAB and those without inoculation of *B*. *glumae* were included as controls. Disease severity on the rice panicles was rated 10 days after inoculation of *B*. *glumae* 336gr-1, using a 0 to 9 scale scheme [80]. Each error bar indicates standard error from three replications and the letters above individual columns indicate significant differences with *P* < 0.05 according to Dunn’s test.

The five RABs selected were further tested for their biological control efficacies on sheath blight and bacterial panicle blight in the field condition. All of the five RABs reduced the symptom development of sheath blight in the susceptible cultivar, Bengal, when applied to the rice 24h prior to the pathogen (*R*. *solani*) inoculation (Fig 5A). RAB9 reduced disease development significantly more than other RABs (Fig 5A). Other four RABs also inhibited disease development significantly compared to the water-treated control (Fig 5A). Sheath blight symptoms were not developed in the uninoculated plants (Fig 5A). Similarly, pretreatments of rice with the RABs 24 h prior to the inoculation of *B*. *glumae* resulted in reduced development of bacterial panicle blight symptoms on rice panicles (Fig 5B). All the five RABs caused significant levels of reduction in bacterial panicle blight symptom development compared to the water-treated control, but there was no significant difference among the RABs tested (Fig 5B).

However, the disease suppression activities of the RABs were significantly reduced in the repeated field trial conducted in the following year (S1 Fig). Only RAB6 and RAB9 showed significant reduction of sheath blight in the repeated trial (S1A Fig), and none of the five RABs retained the disease suppression activities on bacterial panicle blight until the following year (S1B Fig). In a retest of antagonistic activity conducted at four years after the first test, the RABs showed substantial reduction in growth inhibition activity against both *R*. *solani* and *B*. *glumae*, indicating their diminished activities during the long-term storage at -70C (S2 Fig).

### Growth-Promoting Activities of the Selected Antagonistic RABs

The growth-promoting activities of the RABs on rice were determined with the bacterial stocks stored at -70C for four years. Rice seeds were treated with each of the five RABs and then grown in the greenhouse for four weeks. The size of rice seedlings was measured and compared based on the length of shoots and roots. As shown in S3 Fig, RAB9 and RAB18 showed a significant growth-promoting activity for roots and shoots, respectively (S3 Fig).

## Discussion

In this study, 26 RABs having various antifungal and antibacterial activities against *R*. *solani* and *B*. *glumae*, respectively, were isolated from rice plants, and all of them were identified to be *Bacillus* spp. based on their 16S rDNA sequences. Regarding their antagonistic activities against the fungal and bacterial rice pathogens, these RABs can be potential biological agents for both sheath blight and bacterial panicle blight and possibly other plant diseases caused by fungal and bacterial pathogens. Indeed, five RABs showing highest antimicrobial activities (RAB6, RAB9, RAB16, RAB17S and RAB18) were effective in suppressing the development of sheath blight and bacterial panicle blight in the field condition if they were sprayed to rice plants prior to pathogen inoculation.

This study suggests that *Bacillus*-type bacteria are predominant in rice plants as antagonists against pathogens (Table 1). *Bacillus* spp. have been predominantly used to control various crop diseases [29] [30]. Recent examples include citrus canker caused by *Xanthomonas axonopodis* pv. *citri* [31], bacterial wilt and late blight in tomato caused by *Ralstonia solanacearum* and *Phytophthora infestans*, respectively [32–34], bacterial blight in rice caused by *Xanthomonas oryzae* pv. *oryzae* [35], and wheat root rot caused by *Fusarium graminearum* [36]. Specifically, majority of the antagonistic RABs in this study (12 out of 26 RABs) appeared to be *B*. *amyloliquefaciens* based on the nucleotide sequences of 16S rDNA and *gyrB* (Table 1)(Fig 3A), even though this identification is still inconclusive due to the closer similarity of their *recA* sequences to *B*. *subtilis* (Fig 3B). Strains of *B*. *amyloliquefaciens* have been reported as a good biological control agent for other crop diseases, including bacterial wilt and powdery mildew of tomato [34, 37], stem rot of canola [38], bacterial wilt of peanut [39], ring rot of apple [40], and bacterial soft rots of vegetables [41].

The observed antagonistic activities of the RABs appeared to be *B*. *amyloliquefaciens* are likely caused by antimicrobial secondary metabolites. Strains of *B*. *amyloliquefaciens* have been known to produce a variety of antimicrobial compounds. Lipopeptides and cyclic lipopeptides produced by non-ribosomal peptide synthetases (NRPSs), such as surfactin, fengycin, bacillbactin, and bacillomycin D, are thought to be major antimicrobial components of *B*. *amyloliquefaciens* [42]. Additional antimicrobial compounds known to be synthesized non-ribosomally by *B*. *amyloliquefaciens* include polyketides, such as macrolactin and difficidin, and the dipeptide, bacilysin [43, 44]. Bacteriocins produced by *B*. *amyloliquefaciens*, such as subtilosin, amylolysin and amylocyclicin, are also known to suppress phytopathogenic bacteria [45–47]. Nevertheless, it is also possible that direct antimicrobial activities of the RABs may not be solely responsible for their biological control activities against SB and BPB in the field. Instead, induction of plant defense systems by the RABs can be the main mechanism underlying the biological control activities observed in this study. Induction of plant defense systems by *B*. *amyloliquefaciens* has been revealed by several research groups [42, 48–51]. Related to this notion, it was reported that *B*. *amyloliquefaciens* subsp. *plantarum* FZB42, an effective commercial biocontrol agent, did not change significantly the rice root microbiome, but could compensate partially the changes in microbial community structure caused by the pathogen, *R*. *solani* [52]. More comprehensive and integrated studies, incorporating the expression profiles of rice defense-related genes and the microbial communities surrounding rice plants resulted from the treatment of RABs, would lead to better understanding of the mechanisms underlying the biological control activities of the RABs.

Meanwhile, seven antagonistic RABs appeared to be *B*. *methylotrophicus*, a recently defined new species [53], and *B*. *subtilis*, respectively, based on their 16S rDNA sequences. Like *B*. *amyloliquefaciens*, strains of *B*. *methylotrophicus* and *B*. *subtilis* have also been reported as effective biological control and/or plant growth promoting agents [53–56]. A recent whole genome sequence analysis revealed the close relatedness between *B*. *amyloliquefaciens* subsp. *plantarum* and *B*. *methylotrophicus* [57]. The antagonistic RABs closest to *B*. *methylotrophicus* and *B*. *subtilis* are also being investigated for the mechanisms underlying their antimicrobial and biological control activities.

All the three RABs that did not show observable antagonistic activities were closest to one of two *Lysnibacillus* spp., *L*. *sphaericus* (RAB1 and RAB12) or *L*. *xylanilyticus* (RAB5), in 16S rDNA sequence. At this point, it remains to be tested if these RABs have any beneficial activities to rice plants, such as suppression of pathogens and promotion of plant growth, even though none of them showed significant antimicrobial activity against *R*. *solani* and *B*. *glumae in vitro*. So far, information about biological control activities of *Lysinibacillus* spp is relatively limited. A strain of *L*. *fusiformis* isolated from citrus roots was shown to reduce the population of viable *Ca*. L. asiaticus in citrus leaves [58] and some bacteria belonging to this genus have been identified as plant growth-promoting endophytic bacteria [59, 60]. Meanwhile, several strains of *Lysinibacillus* sp. closest to *L*. *sphaericus* and *L*. *xylanilyticus* were shown to have antimicrobial activities against foodborne bacterial and fungal pathogens possibly through bacteriocins [61] and some strains of *L*. *sphaericus* have toxic activities against the larvae of Culex mosquitoes, which are conferred at least in part by S-layer proteins [62] and Mtx toxins [63]. *L*. *sphaericus* was also reported as a potential cellulolytic agent for fermentation of rice straw [64]. It is noteworthy that the binary toxic Bin protein produced by *L*. *sphaericus* showed anticancer activities against human cancer cell lines in a recent study [65].

Subjects of further research on the RABs identified in this study include testing of their antagonistic activities against various pathogens causing other important plant (and possibly animal) diseases and identification and characterization of molecular and/or genetic components responsible for the antimicrobial activities of the RABs to elucidate their underlying mechanisms. In addition, significant reduction of antagonistic and biocontrol activities during the long-term storage at -70C was observed with the RABs in this study, indicating the importance of proper preservation of biocontrol agents. Thus, development of optimal formulation methods for long preservation as well as safe and easy application is required for practical utilization of potential biological control agents identified in this study.

## Materials and Methods

### Isolation of RABs

Leaves of rice plants at the 30% heading stage were collected from the rice field in the LSU AgCenter Rice Research Station at Crowley, Louisiana. The collected leaves were cut into ~ 4 cm-long pieces and subsequently washed by stirring in 500 ml of sterilized ddH_2_O for 10 min or in 500 ml of 10% bleach for 5 min. The bleach-sterilized leaf pieces were then stirred in sterilized ddH_2_O for 10 min to remove the remaining bleach. The washed leaf pieces were placed on potato dextrose agar (PDA) plates, making the adaxial side contact to the medium, and incubated at room temperature for 72 h. Bacterial colonies grown out from the leaf samples were transferred to new PDA plates for pure isolation of RABs.

### Measurement of RABs’ Antimicrobial Activities against *R*. *solani* and *B*. *glumae*

To determine antifungal activities of RABs against *R*. *solani*, a mycelial plug of *R*. *solani* was taken from one-week-old culture of the fungal pathogen on potato dextrose agar (PDA) using a cork borer (5 mm in diameter) and placed on the center of a fresh PDA plate. Each RAB was cultured overnight in L broth (LB) (10 g tryptone, 10 g NaCl and 5 g yeast extract per L) in a shaking incubator at 37C at 190 rpm. The 1.5 ml of each culture was then washed twice with fresh LB and resuspended in 100 μl of LB. Ten μl of the bacterial suspension was spotted on three locations around the mycelial plug on PDA. Observation of antifungal activities and measurement of inhibition zones were conducted 72 h after incubation at 25C.

To determine antibacterial activities of RABs against *B*. *glumae*, *B*. *glumae* strain 336gr-1 was cultured overnight in LB at 37C and the overnight culture was washed twice with fresh LB. One hundred microliters of the bacterial suspension adjusted to OD_600_ = 0.1 (ca. 5×10^7^ CFU/ml) was spread-plated on a PDA plate. Bacterial suspensions of RABs were prepared as described above and 10 μl of each sample was spotted on three location of a PDA plate previously spread with *B*. *glumae*. Observation of antibacterial activities and measurement of inhibition zones were conducted 72 h after incubation at 25C.

### Identification of the Antagonistic RABs

The Ryu’s 3% KOH test [66, 67] was performed to initially categorize RABs into Gram-positive or Gram-negative bacteria. *B*. *glumae* and *Bacillus subtilis* were used as a control representing Gram-negative and Gram-positive bacteria, respectively. For sequencing of 16S rDNA, genomic DNA of each RAB was extracted using a previously described method [68] and 16S rDNA sequence was amplified using the primers fD1 (5’CCGAATTCGTCGACAACAGAGTTTGATCCTGGCTCAG3’) and rD1 (5’CCCGGGATCCAAGCTTAAGGAGGTGATCCAGCC3’) [69]. Each reaction of PCR contained 3 μl of genomic DNA (~ 100 ng/μl), 2.5 μl of 10X PCR buffer, 0.75 μl of 50mM MgCl_2_, 0.5 μl of 10mM dNTP mix, 1.0 μl of homemade *Taq* polymerase (~ 1.0U/μl), 1 μl of 10 μM forward (fD1) and reverse (rD1) primers, and 15.25 μl of sterilized ddH_2_O in a total volume of 25 μl. The PCR program consisted of the initial denaturation at 95°C for 1 min; 35 cycles of 95°C for 2 min, 42°C for 30 sec and 72°C for 4 min; and the final extension at 72°C for 20 min. For sequencing *gyrB*, primers UP-1 (5’ GAAGTCATCATGACCGTTCTGCAYGCNGGNGGNAARTTYGA 3’) and UP-2r (5’ AGCAGGGTACGGATGTGCGAGCCRTCNACRTCNGCRTCNGTCAT 3’) were used for *gyrB* gene amplification, and UP-1S (5’ GAAGTCATCATGACCGTTCTGCA 3’) and UP-2Sr (5’ AGCAGGGTACGGATGTGCGAGCC 3’) were used for sequencing [70]. For sequencing *recA*, primers *rec*A-F (5' TGAGTGATCGTCAGGCAGCCTTTAG 3') and *rec*A-R (5' CYTBRGATAAGARTACCAWGMACCGC 3') were used for both gene amplification and sequencing [71]. Each reaction for PCR has a total volume of 25 μl containing 2 μl of ~100 ng/μl of genomic DNA as a template, 2.5 μl of 10X PCR buffer (Agilent Technologies, Inc., Santa Clara, CA), 0.5 μl of 10 mM dNTPs, 0.2 μl of 5 U/μl of Paq5000 DNA polymerase (Agilent Technologies, Inc., Santa Clara, CA), 1 μl of 10 μM of forward and reverse primers, and 17.8 μl of sterilized ddH_2_O. The PCR program for both *gyrB* and *recA* genes was set to initial denaturation at 95°C for 3 min followed by 30 cycles of 94°C for 1 min, 60°C for 1 min, 72°C for 2 min, and the final extension of 72°C for 10 min.

PCR products were purified using a QuickClean 5M PCR Purification Kit (GenScript, Piscataway, NJ) and sent to Macrogen Inc. (Seoul, Korea) for sequencing. Sequence results were assembled using a software SeqTrace [72], aligned and cured using MUSCLE [73] and Gblocks [74, 75], respectively, and searched against the National Center for Biotechnology Information (NCBI) database to identify the corresponding or homologous sequences, using Basic Local Alignment Search Tool (BLAST). Phylogenetic analysis with the DNA sequence data of 16S rDNA, *gyrB* and *recA* was performed with neighbor joining method using MEGA 5 [76].

### Evaluation of RABs’ Inhibitory Activities on Sclerotial Germination of *R*. *solani*

The effects of RABs on sclerotial germination of *R*. *solani* were observed following a previous method [7] with some modifications. Briefly, young and fresh sclerotia collected from mycelia of *R*. *solani* grown on PDA were surface-sterilized with 2% sodium hypochlorite solution for 2 min and washed with sterilized ddH_2_O. The surface-sterilized sclerotia were put in the overnight-grown cultures of RABs (> 1 X 10^9^ CFU/ml) and further incubated in a shaking incubator for 24 h at 25C at 200 rpm. The sclerotia incubated in a RAB culture were then gently taken out and placed on fresh PDA plates. Germination rate of sclerotia was determined after 72 h of incubation at 25C.

### Evaluation of the RABs’ Inhibitory Activities on the Lesion Development by *R*. *solani* on Detached Rice Leaves

The detached leaf assay to examine the inhibition of sheath blight lesion development by each RAB was performed following a previous method [77] with minor modifications. Briefly, the second leaf from the base was taken from a two-month-old rice plant of the disease susceptible cultivar, Bengal, and cut into ~ 6 cm-long pieces. The leaf pieces were surface-sterilized with 1% sodium hypochlorite solution for 1 min and washed with sterilized ddH_2_O. The sterilized leaf pieces were then placed on petri plates containing a wet filter paper, and were pressed with sterilized slide glasses to keep the leaves flat during the experiment. Overnight grown RAB culture in LB broth in a shaking incubator at 25C at 200 rpm was washed two times in a fresh LB, and resuspended in sterilized ddH_2_O, adjusting the RAB concentration to *ca*. 6×10^8^ CFU/ml. Each RAB cell suspension was spread on leaf surfaces with a sterile cotton swab. A sclerotium collected from the one-week-old mycelia of *R*. *solani* was placed on the center of each leaf piece. Three leaf pieces were treated with each RAB for three replications. Leaf pieces without any RAB treatment and those treated with sterilized ddH_2_O were also included as controls. The petri dishes containing rice leaf pieces placed with a screlotium were incubated at 25C for 7 days with 12 h-light period per day. The relative lesion length on a detached leaf piece was calculated using the following way according to [78]:

$$Relative\;lesion\;length\;(\%)=[\frac{Lesion\;length}{\left(Leaf\;length\right)}]X\;100$$

### Evaluation of the RABs’ Biocontrol Activities on Sheath Blight and Bacterial Panicle Blight

#### Treatment of rice plants with RABs

The medium-grain and disease susceptible cultivar, Bengal, was grown in the field of the Rice Research Station (Crowley, Louisiana) in 2012. Approximately 15 rice plants grown in a row were treated with each RAB (or water for negative control) and overall disease severity of each row was recorded. Data of six replications were obtained from each treatment for statistical analysis. Overnight cultures of RAB grown on LB agar were resuspended in deionized water and bacterial suspension of *ca*. 5 X 10^7^ CFU/ml (OD_600_ = 0.1) of each RAB was sprayed onto rice panicles until it drips.

#### Inoculation of rice plants with *R*. *solani*

The inoculum of *R*. *solani* was prepared in a mixture of rice husk and grain. Briefly, 600 g of the rice husk/grain mixture (husk 2: grain 1 w/w) were moisturized with 500 ml of water in an 1 L-flask and sterilized at 121C for 20 min. The sterilized mixture in a flask was inoculated with a *ca*. 16 cm^2^ PDA plug containing 7 day-old *R*. *solani* mycelia, and incubated at 25C for 10 days. After 10 days of incubation the prepared inoculum was mixed with a fresh sterilized rice husk/grain mixture with 1:2 ratio of prepared inoculum and fresh mixture to increase the inoculum volume. After mixing thoroughly, the mixture was spread uniformly on a clean brown paper sheet and covered with a clean plastic sheet at room temperature. After 24 h of incubation at room temperature, the prepared *R*. *solani* inoculum was applied to rice plants at the tillering stage by hand sprinkling at 24 h post treatment of RAB. The symptoms were observed 27 days after inoculation (at the milk stage of rice). Disease severity was rated based on relative lesion size with the scale ranges from 0 to 9 [79].

#### Inoculation of rice plants with *B*. *glumae*

*B*. *glumae* 336gr-1 was grown overnight on King’s B agar at 37°C and the bacterial cells were resuspended in deionized water, adjusting the concentration to ~1×10^8^ CFU/ml. The bacterial suspension was sprayed onto rice panicles at the 30% heading stage at 24 h post treatment of each RAB. The symptoms were observed 10 days after inoculation. Disease severity was rated based on discolored area and sterility of panicles with the scale ranges from 0 to 9 [80].

### Evaluation of Rice Growth-Promoting Activities of RABs

Rice seeds (cv. Trenasse) were surface-sterilized with 2.5% sodium hypochlorite for 3 min and washed three times with sterile ddH_2_O. RABs were grown overnight in L broth overnight at 37C in a shaking incubator at 190 rpm. Overnight grown RAB cultures were washed twice with fresh L broth, and resuspended in sterile ddH_2_O adjusting to OD_600_ = 0.2 (~ 1 × 10^7^ CFU/ml). The surface-sterilized rice seeds were soaked in the five different RAB suspensions or in sterile ddH_2_O for control, and incubated for 24 h at room temperature on a shaker. After incubation, four replications per RAB with 10 rice seeds in each replication were grown in a mixture of sterile soil and sand (3:1 ratio) in 4.5 inch-diameter pots in the greenhouse. The length of shoot and root were measured 4 weeks after planting. Shoot length of the rice seedlings was measured from the tip of the plant to its base, and root length was measured from the base to the root tip.

## Supporting Information

S1 Fig Disease suppression by the five RABs measured in the repeated field trial in the following year (2013). (A) Suppression of sheath blight by pretreatment of RABs 24 h prior to the inoculation of *Rhizoctonia solani* during the tillering stage of Trenasse. Disease score was recorded based on a 0–9 scale. Different lowercase alphabets indicate significant differences with p < 0.05 according to Dunn’s test. (B) Suppression of bacterial panicle blight by pretreatment of RABs 24 h prior to the inoculation of *Burkholderia glumae* 336gr-1 at the 30% heading stage of Trenasse. Disease score was recorded based on a 0–9 scale. Different lowercase alphabets indicate significant differences with p < 0.05 according to Dunn’s test.
(TIF)

S2 Fig Diminished antagonistic activities of RABs. (A) Antifungal activities of RABs against *Rhizoctonia solani*. (B) Antibacterial activities of RABs against *Burkholderia glumae*. The blue and orange columns indicate the experimental data in 2011 and 2015, respectively. Each error bar indicates standard deviation from three replications.
(TIF)

S3 Fig Growth-promoting activity of RABs. Rice seeds were incubated with individual RABs (or with sterile ddH_2_O for ‘control’) for 24 h at room temperature in a shaker at 100 rpm. After incubation seeds were dried, sown in a sterilized soil and sand mixture, and grown in the greenhouse. Rice seedlings were measures at 4 weeks after sowing. Different alphabets on the top of each bar indicate significant difference at α = 0.05. Each error bar indicates standard error from four replications.
(TIF)
