# Supplementary figures and images for: Biological Control Activities of Rice-Associated Bacillus sp. Strains against Sheath Blight and Bacterial Panicle Blight of Rice
Source: PLoS One. 2016 Jan 14;11(1):e0146764. doi: 10.1371/journal.pone.0146764 (PMC4713167; doi:10.1371/journal.pone.0146764)

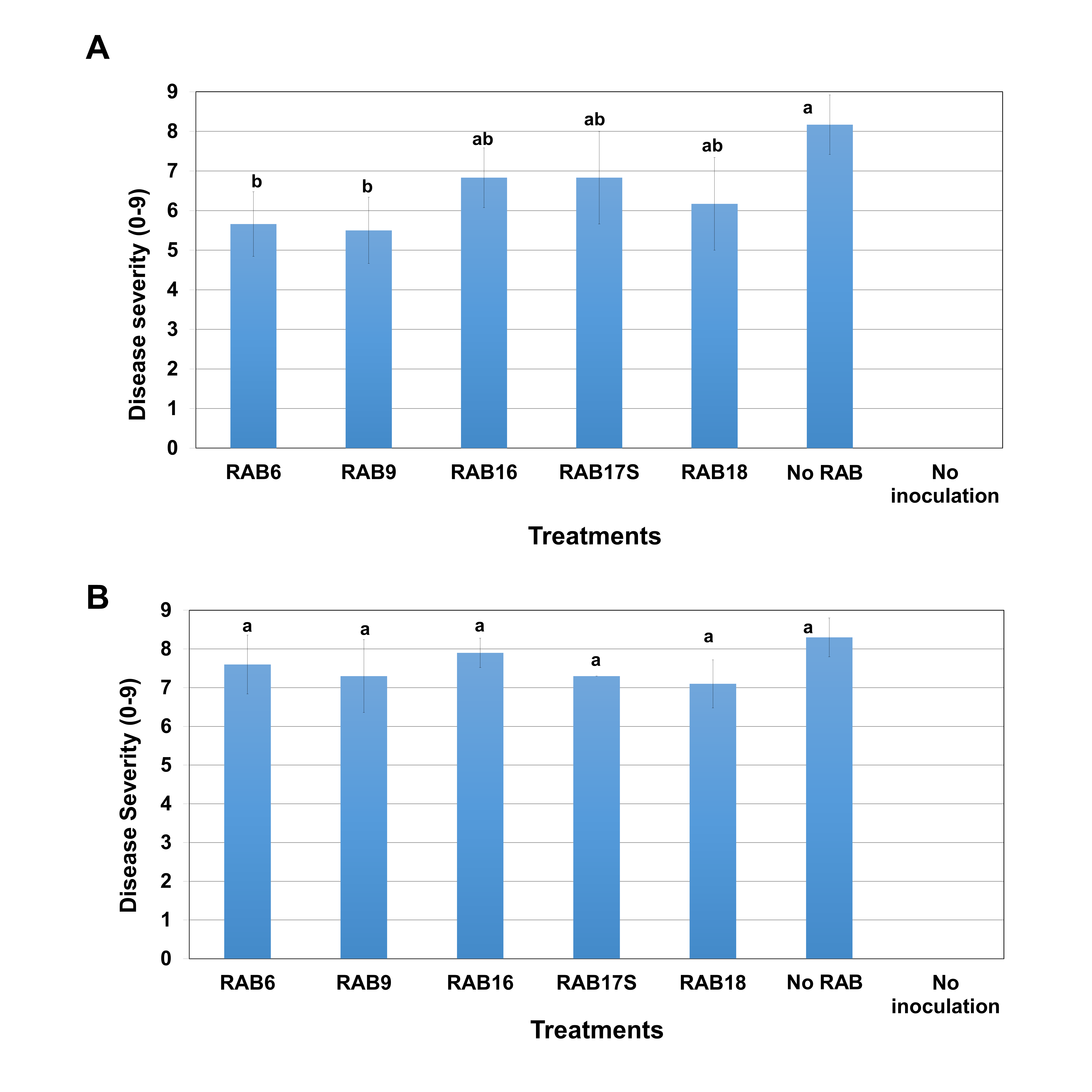

Supplement: S1 Fig — (A) Suppression of sheath blight by pretreatment of RABs 24 h prior to the inoculation of Rhizoctonia solani during the tillering stage of Trenasse. Disease score was recorded based on a 0–9 scale. Different lowercase alphabets indicate significant differences with p < 0.05 according to Dunn’s test. (B) Suppression of bacterial panicle blight by pretreatment of RABs 24 h prior to the inoculation of Burkholderia glumae 336gr-1 at the 30% heading stage of Trenasse. Disease score was recorded based on a 0–9 scale. Different lowercase alphabets indicate significant differences with p < 0.05 according to Dunn’s test. (TIF) [file pone.0146764.s001.tif]

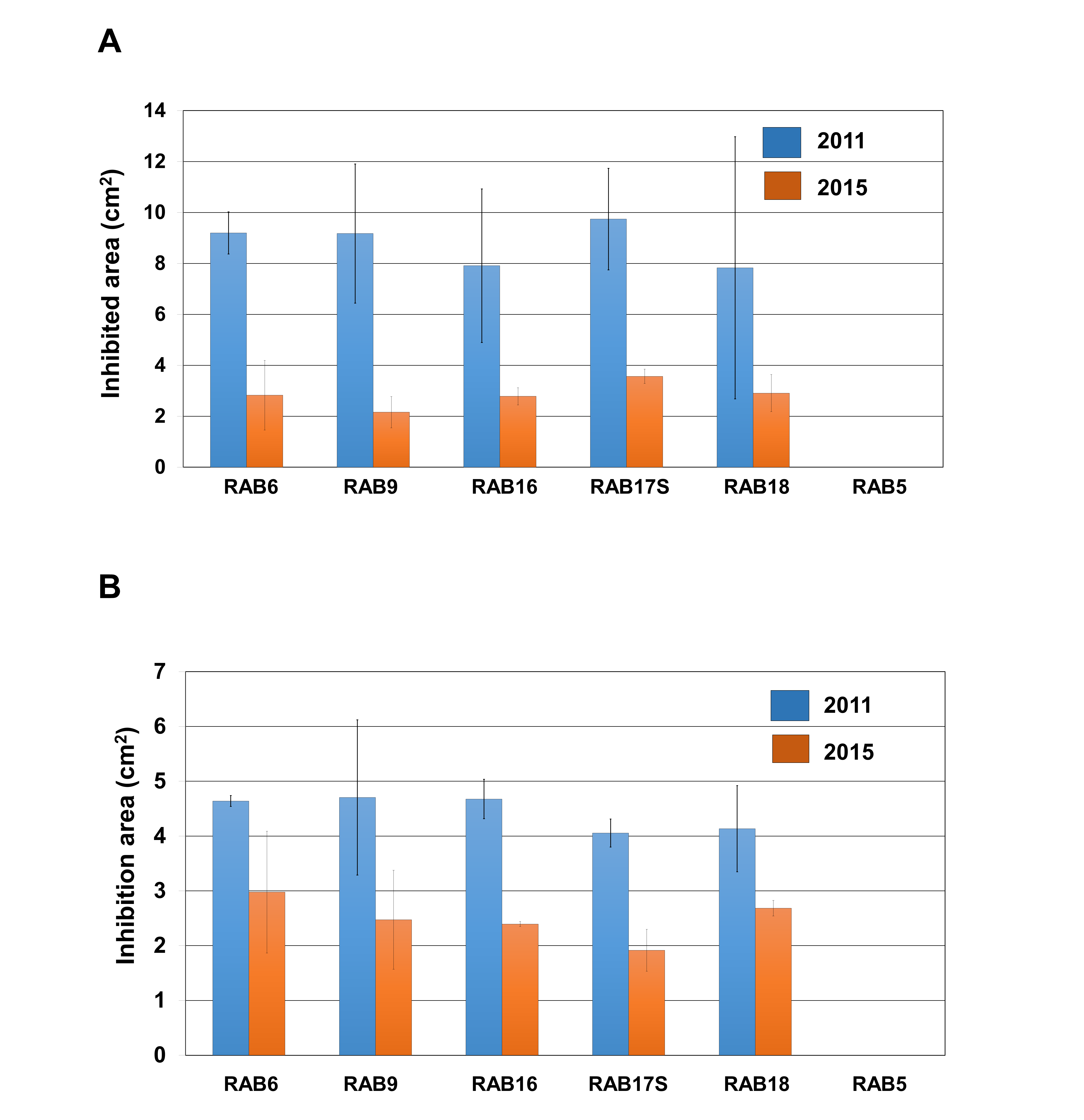

Supplement: S2 Fig — (A) Antifungal activities of RABs against Rhizoctonia solani. (B) Antibacterial activities of RABs against Burkholderia glumae. The blue and orange columns indicate the experimental data in 2011 and 2015, respectively. Each error bar indicates standard deviation from three replications. (TIF) [file pone.0146764.s002.tif]

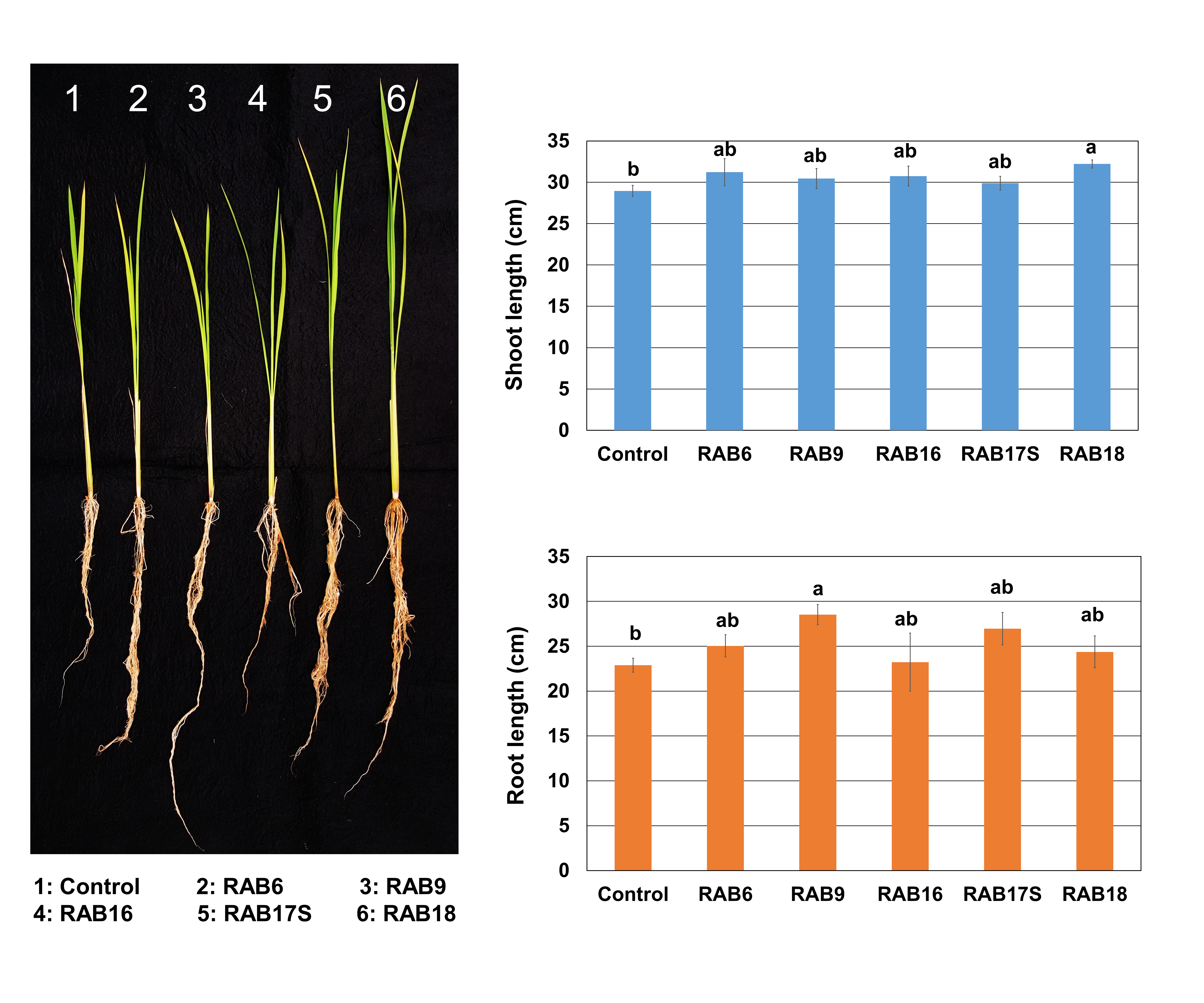

Supplement: S3 Fig — Rice seeds were incubated with individual RABs (or with sterile ddH2O for ‘control’) for 24 h at room temperature in a shaker at 100 rpm. After incubation seeds were dried, sown in a sterilized soil and sand mixture, and grown in the greenhouse. Rice seedlings were measures at 4 weeks after sowing. Different alphabets on the top of each bar indicate significant difference at α = 0.05. Each error bar indicates standard error from four replications. (TIF) [file pone.0146764.s003.tif]
